# Combined HPV 16 E2 and L1 methylation predict response to treatment with cidofovir and imiquimod in patients with vulval intraepithelial neoplasia

**DOI:** 10.3233/CBM-210448

**Published:** 2022-10-12

**Authors:** Christopher Nicholas Hurt, Belinda Nedjai, Carlos Alvarez-Mendoza, Ned Powell, Amanda Tristram, Sadie Jones

**Affiliations:** a Centre for Trials Research, Cardiff University, Cardiff, UK; b Wolfson Institute of Preventive Medicine, Centre for Cancer Prevention, Queen Mary University of London, London, UK; c Queensland Brain Institute, The University of Queensland, Brisbane, Australia; d Centre for Medical Education, Cardiff University, Cardiff, UK; e Wellington Regional Hospital, Wellington, New Zealand; f School of Medicine, Cardiff University, Cardiff, UK

**Keywords:** Vulval intraepithelial neoplasia, HPV, gene methylation, predictive biomarker, cidofovir, imiquimod

## Abstract

**BACKGROUND:**

Topical cidofovir and imiquimod can effectively treat approximately 55% of patients with vulval intraepithelial neoplasia (VIN), thus avoiding the need for surgery. Human papillomavirus (HPV) E⁢2 gene methylation predicts response to treatment but a methylation measurement is only obtainable in approximately 50% of patients.

**OBJECTIVE:**

This work aimed to determine if the applicability and predictive power of the E⁢2 methylation assay could be improved by combining it with the components of a host and viral DNA methylation panel (S5) that has been found to predict disease progression in patients with cervical intraepithelial neoplasia.

**METHODS:**

HPV E2 methylation and S5 classifier score were measured in fresh tissue samples collected pre-treatment from 132 patients with biopsy-proven VIN grade 3 who participated in a multicentre clinical trial and were randomised to treatment with cidofovir or imiquimod.

**RESULTS:**

Combining HPV16 E⁢2 and HPV16 L⁢1 methylation provides a biomarker that is both predictive of response to topical treatment and that can produce a clinically applicable result for all patients. Patients with HPV 16 $L\,1^{\text{ℎ𝑖𝑔ℎ}}$**and** HPV 16 $E\,2^{\text{ℎ𝑖𝑔ℎ}}$ (36/132 (27.3%)) were more likely to respond to treatment with cidofovir (12/15 (80.0%)) than imiquimod (9/21 (42.9%)) (p= 0.026). Patients with HPV 16 $L\,1^{\text{𝑙𝑜𝑤}}$**or** HPV 16 $E\,2^{\text{𝑙𝑜𝑤}}$ (including those with no HPV/unassessable methylation) were more likely to respond to imiquimod: 23/50 (46.0%) vs 31/46 (67.4%) (p= 0.035).

**CONCLUSIONS:**

Combined HPV E⁢2 and L⁢1 methylation is a potential predictive marker in treatment for all patients with VIN. These findings justify validation in a prospective trial.

## 1. Introduction

Vulval intraepithelial neoplasia (VIN) is a chronic condition of vulval skin and diagnosis is confirmed histologically by the identification of cellular changes associated with a premalignant state. VIN is commonly caused by human papillomavirus (HPV), which is present in around 76% of cases [1]. VIN can be very distressing for patients and often takes a long time to diagnose. If untreated, VIN may progress to vulval cancer.

Surgical excision has historically been the gold standard of care for patients with VIN but is associated with significant morbidity [2, 3]. With an increasingly younger population of women being affected by VIN, and recurrence rates in the region of 40–60% [4, 5], optimised alternative treatments are urgently needed. In a recent randomised trial (RT3VIN), topical treatment with imiquimod (an immune response modulator) versus cidofovir (a cytotoxic cytosine analogue specially formulated for topical treatment) both demonstrated complete response rates of 46% [6]. Both these treatments are challenging for patients, causing pain, irritation and ulceration, leading to premature discontinuation of treatment in up to 26% of patients. However, in patients who respond, recurrence rates appear to be lower than the recurrence rates seen in patients treated surgically and were lower with cidofovir compared to imiquimod (6% vs 28% at 18 months) [7]. The ability to identify patients more likely to respond to these treatments prior to their commencement could help to motivate the patient to persevere with the treatment and reassure the clinician that the right treatment is being offered.

Previous research demonstrated that HPV 16 E⁢2 methylation has the potential to serve as a predictive biomarker in the context of topical treatment of VIN [8]. Patients with higher levels of HPV E⁢2 methylation were shown to be more likely to respond to treatment with cidofovir, and patients with lower levels of HPV E2 methylation were more likely to respond to treatment with imiquimod. An E⁢2 methylation level of > 4% predicted response to treatment with cidofovir with 88.2% sensitivity and 84.6% specificity, while < 4% E⁢2 methylation predicted response to treatment with imiquimod with 70.6% sensitivity and 62.5% specificity. Clinical application of such a biomarker could enable more efficient treatment for patients with VIN, lower rates of recurrence and reduced reliance on surgery for effective treatment thereby avoiding the morbidity associated with surgical excision.

The potential of HPV E⁢2 methylation as a predictive biomarker was limited by the HPV E⁢2 methylation result being unobtainable for 68/132 (52%) patients. Factors contributing towards this include the absence of HPV 16 in a significant number of patients (26/132 (20%)), disruption of the E⁢2 gene (a common step in HPV pathogenesis) [9, 10], and poor quality/low levels of DNA in the samples. To develop a biomarker with clinical utility, these areas need to be addressed.

The S5 classifier is a DNA methylation panel including both human and HPV DNA sequences that has been validated as a triage test in the detection of CIN and cervical cancer [11]. Additionally, it and has been shown to have high potential as a prognostic biomarker to accurately identify progressive cervical intraepithelial neoplasia 2 (CIN 2) [12]. The S5 classifier targets the late (L1 and/or L2) viral regions of HPV 16, 18, 31 and 33 as well as host gene *EPB41L3*. This assay has several potential advantages: it is not reliant on HPV positivity to generate a result (due to inclusion of the EPB41L3 host gene), it detects methylation in three additional HPV genotypes and targets a region (L⁢1⁢L⁢2) of viral DNA that is potentially less likely to be affected by viral integration [10], and a result is generated for all samples (as missing methylation values are imputed as zero).

The aims of this work were to:

• determine whether or not the HPV 16 E⁢2 methylation assay failure rate could be improved and results replicated in a second laboratory
• determine whether or not the S5 classifier or any of its constituent parts could predict response to treatment
• explore combining the S5 classifier or any of its constituent parts with HPV 16 E⁢2 methylation to further improve response prediction.

## 2. Materials and methods

### 2.1 Patients, samples and DNA extraction

This study utilised a subset of bioresources from the RT3VIN clinical trial which included women who were age 16 years or older and had biopsy-proven VIN grade 3 (including visible perianal disease not extending into the anal canal) within the past 3 months. Baseline translational research samples were available in addition to the clinical outcome data (n= 132) [6]. 4 mm punch biopsies were taken at baseline (prior to treatment) adjacent to the biopsy in which VIN 3 was confirmed histologically. The biopsies were stored in ThinPrep media (Hologic) prior to processing. DNA was extracted using the Qiagen DNA Mini Kit (Qiagen). The DNA extraction, HPV detection and initial HPV 16 E⁢2 methylation analysis (described below) were performed in the HPV Research Laboratory at Cardiff University between 2010 and 2014. The later E⁢2 methylation and S5 classifier analysis (described below) was carried out at Queen Mary’s University London (QMUL) in 2019/2020.

Consent for sample collection and their use in research was obtained from patients when they were recruited into the RT3VIN trial and was approved by the UK Multi Research Ethics Committee (08/NIR03/82).

### 2.2 HPV detection 

A type-specific PCR targeting the HPV 16 E⁢6 region [13] was used to detect cases of HPV 16. The Greiner PapilloCheck HPV genotyping assay (Greiner Bio-One), which tests for 24 HPV genotypes (HPV 6, 11, 40, 42, 43, 44, 16, 18, 31, 33, 35, 39, 45, 51, 52, 53, 56, 58, 59, 66, 68, 70, 73, and 82), was used as per manufacturer’s instructions to test for the presence of additional genotypes.

### 2.3 Methylation analysis in the Cardiff University HPV research laboratory (CU HPV Lab)

HPV DNA methylation testing was restricted to HPV16 positive (n= 106) cases, and DNA methylation was quantified in the E⁢2 region only. The laboratory was blinded to treatment response. Positioning of primer sequences reflected sequence constraints and the desire to amplify the maximum number of CpG sites within a single reaction. DNA (500 ng) was sodium bisulfite treated using the EZ-DNA Methylation Kit (Zymo Research Corp). DNA methylation was assessed by pyrosequencing of the E⁢2 ORF using a Qiagen PyroMark Q96 ID system as previously described [14]. The assay targeted multiple CpGs (3411, 3414, 3416, 3432, 3435, and 3447) and methylation levels were reported as means for each region. Stringent quality assurance checks were applied to the methylation data, including assessment of bisulphite conversion and primer extension; additional quality control assessments were performed by the pyrosequencing software, and any sample classed as “fail” was excluded from the analysis. All samples were run in duplicate and the standard deviation was calculated for each CpG site analyzed; samples were excluded from further analysis if a value was beyond 3 standard deviations of the mean standard deviation calculated for all CpG sites for each region.

### 2.4 Methylation analysis in Queen Mary’s University of London laboratory (QMUL Lab)

All 132 baseline samples were tested for the components of the S5 classifier as well as HPV16 E⁢2 and the laboratory was blinded to the treatment response. The procedures and quality control of these experiments have been reported previously [12, 15]. In brief, DNA was quantified with Qubit High Sensitivity and Broad Range Kits (Thermo Fisher). Bisulfite conversion was carried out on 250 ng of DNA with a volume of 20 ul of elution buffer using the EZ DNA Methylation Kit (Zymo Research Corp). After DNA conversion, the PyroMark PCR Kit (Qiagen) was used to amplify the following CpG sites: HPV 16 L⁢1 (6367 and 6389), HPV 16 L⁢2 (4238, 4259, and 4275), HPV 16 E⁢2 (same as Cardiff above), HPV 18 L⁢2 (4256, 4261, 4265, 4269, 4275, and 4282), HPV 31 L⁢1 (6352, 6364), HPV 33 L⁢2 (5557, 5560, 5566, and 5572), and human gene *EPB41L3* (425, 427, and 438). PCR products were pyrosequenced using a PyroMark Q96 ID instrument (Qiagen). The pyrosequencing software (Pyromark CpG Qiagen) returns a methylation value (%) and a quality value for each CpG in the sequence; “failed samples” were treated as “missing”, “check samples” were compared with the amplitude of the control results and included if satisfactory.

### 2.5 Statistical methods

Statistical analyses were conducted using STATA/SE v16 according to a pre-specified analysis plan. The dataset used in this analysis included all patients from the RT3VIN trial who had both a pre-treatment tissue sample and a post-treatment histological assessment response available.

**Table 1:** Distribution of DNA methylation factors in baseline samples

	Cidofovir${}^{*}$				Imiquimod${}^{*}$			
Randomised	89				91			
with response endpoint	72 (80.9)				71 (78.0)			
and baseline sample	65 (73.0)				67 (73.6)			
Analysis dataset	65				67			
Unifocal VIN	32 (49.2)				33 (49.3)			
Multifocal VIN	33 (50.8)				34 (50.8)			
First VIN	35 (53.9)				36 (53.7)			
Recurrent VIN	30 (46.2)				31 (46.3)			
HPV+ve	59 (90.1)				55 (82.1)			
HPV16+ve	52 (80.0)				54 (80.6)			
	n with a reading		% methylation${}^{**}$ – median (IQR)		n with a reading		% methylation${}^{**}$ – median (IQR)	
EPB41L3	65	(100)	5.3	(3.5–11.9)	67	(100)	4.7	(3.1–7.8)
HPV 16 L⁢1	45	(69.2)	62.5	(22.0–92.8)	47	(70.1)	51.91	(20.0–92.7)
HPV 16 L⁢2	40	(61.5)	11.7	(9.1–82.4)	38	(56.7)	18.2	(9.8–67.8)
HPV 18	1	(1.5)	32.6		0	(0)	–	
HPV 31	0	(0)	–		1	(1.5)	22.64	
HPV 33	8	(12.3)	43.7	(13.8–75.5)	5	(7.5)	46.0	(41.1–64.6)
S5	65	(100)	10.9	(7.3–18.0)	67	(100)	10.5	(6.1–14.9)
HPV 16 E⁢2 (QMUL)	35	(53.8)	4.5	(3.4–42.5)	38	(56.7)	7.8	(4.0–81.6)
HPV 16 E⁢2 (Cardiff)	30	(46.2)	4.3	(2.2–35.6)	34	(50.7)	3.4	(2.4–33.6)

n* (%) unless otherwise shown. ${}^{**}$Only including those with a reading i.e. prior to imputation using zero for missing values.

HPV 16 E⁢2 mean methylation obtained at the CU HPV Lab has been previously described [8]. In this new analysis conducted by the QMUL Lab, mean methylation across CpGs was calculated for: *EPB41L3*, HPV 16 E⁢2, HPV 16 L⁢1, HPV 16 L⁢2, HPV 18 L⁢2, HPV 31 L⁢1, and HPV 33 L⁢1. Any missing values were then imputed as zero as required for calculation of the S5 classifier. As in previous work [14], the mean methylation results (for HPV 16 L⁢2, the proportion of CpGs which show any methylation) were used in the calculation below:

S5 = (30.9 × EPB41L3) + (13.7 × HPV16-L1) + (4.3 × HPV16-L2) + (8.4 × HPV18-L2) + (22.4 × HPV31-L1) + (20.3 × HPV33-L1).

The HPV 16 E2 methylation results were compared between laboratories using a scatter and Bland-Altman plot but all other analyses used the HPV 16 E2 methylation results from the QMUL Lab as it was thought to better represent a routine NHS clinical laboratory.

HPV positivity was defined as any PapilloCheck HPV genotype or HPV16 E⁢6 PCR or any result > 0 for HPV 16 L⁢1, HPV 16 L⁢2, HPV 16 E⁢2, HPV 18, HPV 31, or HPV 33 methylation. HPV16 positivity was defined as any PapilloCheck HPV 16 genotype or E6 PCR or any result > 0 for HPV 16 L⁢1, HPV 16 L⁢2 or HPV 16 E⁢2 methylation.

For each treatment arm, Wilcoxon rank sum tests were used to compare the distribution of each methylation marker, and chi-square tests to compare the proportion with HPV 16/any HPV positivity, between the group who responded to treatment and the group who did not respond to treatment. Multiple comparisons were accounted using the Benjamini-Hochberg procedure [16] by setting the false discovery rate (Q) at 0.1 and calculating each individual p-value’s critical value, using the formula (i/m)Q, where I = the individual p-value’s rank and m = total number of tests. Significant findings where p was less than the critical value for any biomarker in either treatment cohort were further investigated using ROC curve analysis to find optimum cut-offs for sensitivity and specificity. These binary variables were then included in multivariable exact logistic regression analyses of response that also included clinical variables thought to be prognostic of response (disease focality [unifocal/multifocal] and recurrence [first presentation/recurrent]). We used any arising findings to see whether or not we could improve the accuracy of the previously established cut-off of 4% HPV 16 E⁢2 methylation in predicting which treatment may be best for which patients.

In order to examine the validity of the pragmatic imputation of missing methylation values as zero, we conducted post-hoc sensitivity analyses to examine the consistency of findings across subgroups of patients: those with missing methylation and no relevant HPV genotype detected (thought to be most likely to be true zeros) and those with missing methylation but relevant genotype detected (thought to potentially be explained by viral integration and therefore potentially spurious zeros). 

**Table 2:** Distribution of DNA methylation factors in responders and non-responders by trial arm

	Cidofovir (N= 65)						Imiquimod (N= 67)					
	No response (N= 30)		Response (N= 35)		Rank sum result	(i/m) Q${}^{**}$	No response (N= 27)		Response (N= 40)		Rank sum result	(i/m) Q${}^{**}$
*HPV 16*	*n (%)*		*n (%)*				*n (%)*		*n (%)*			
Negative	5	(38.5)	8	(61.5)	$\lambda^{2}=$ 0.387	0.071	3	(23.1)	10	(76.9)	$\lambda^{2}=$ 1.988	0.029
Positive	25	(48.1)	27	(51.9)	p= 0.534		24	(44.4)	30	(55.6)	p= 0.159	
*Any HPV*	*n (%)*		*n (%)*				*n (%)*		*n (%)*			
Negative	2	(50.0)	2	(50.0)	$\lambda^{2}=$ 0.025	0.1	0	(0.0)	4	(100.0)	$\lambda^{2}=$ 2.871	0.021
Positive	28	(45.9)	33	(54.1)	p= 0.873		26	(42.9)	36	(57.1)	p= 0.090	
*Methylation*	Median (IQR)${}^{*}$		Median (IQR)${}^{*}$				Median (IQR)${}^{*}$		Median (IQR)${}^{*}$			
HPV16 E⁢2	2.7	(0–3.7)	2.7	(0–15.7)	z=-0.811 p= 0.418	0.057	3.2	(0–81.6)	2.2	(0–6.6)	z= 1.320 p= 0.187	0.043
S5	9.8	(7.4–16.3)	13.4	(6.7–18.2)	z=-0.868 p= 0.385	0.050	13.4	(8.9–17.7)	2.2	(9.3–13.4)	z= 2.339 p= 0.019	0.014
EPB41L3	5.1	(3.4–11.2)	5.7	(3.5–13.7)	z=-0.632 p= 0.528	0.064	4.8	(2.7–10.4)	4.7	(3.2–5.9)	z= 0.256 p= 0.798	0.093
HPV 16 L⁢1	21.2	(0–85.6)	28.8	(0–92.1)	z=-0.307 p= 0.759	0.079	**36.0**	**(14.4–93.4)**	**12.2**	**(0–55.7)**	z=** 2.714**p= 0.006	0.007
HPV 16 L⁢2	8.1	(0–10.6)	8.1	(0–62.2)	z= 0.273 p= 0.785	0.086	8.9	(0–67.8)	3.2	(0–15.0)	z= 1.340 p= 0.180	0.036

${}^{*}$Missing results treated as “0”. ${}^{**}$Critical value to control for false discovery rate – p must be below this to be counted as significant.

**Figure 1.**
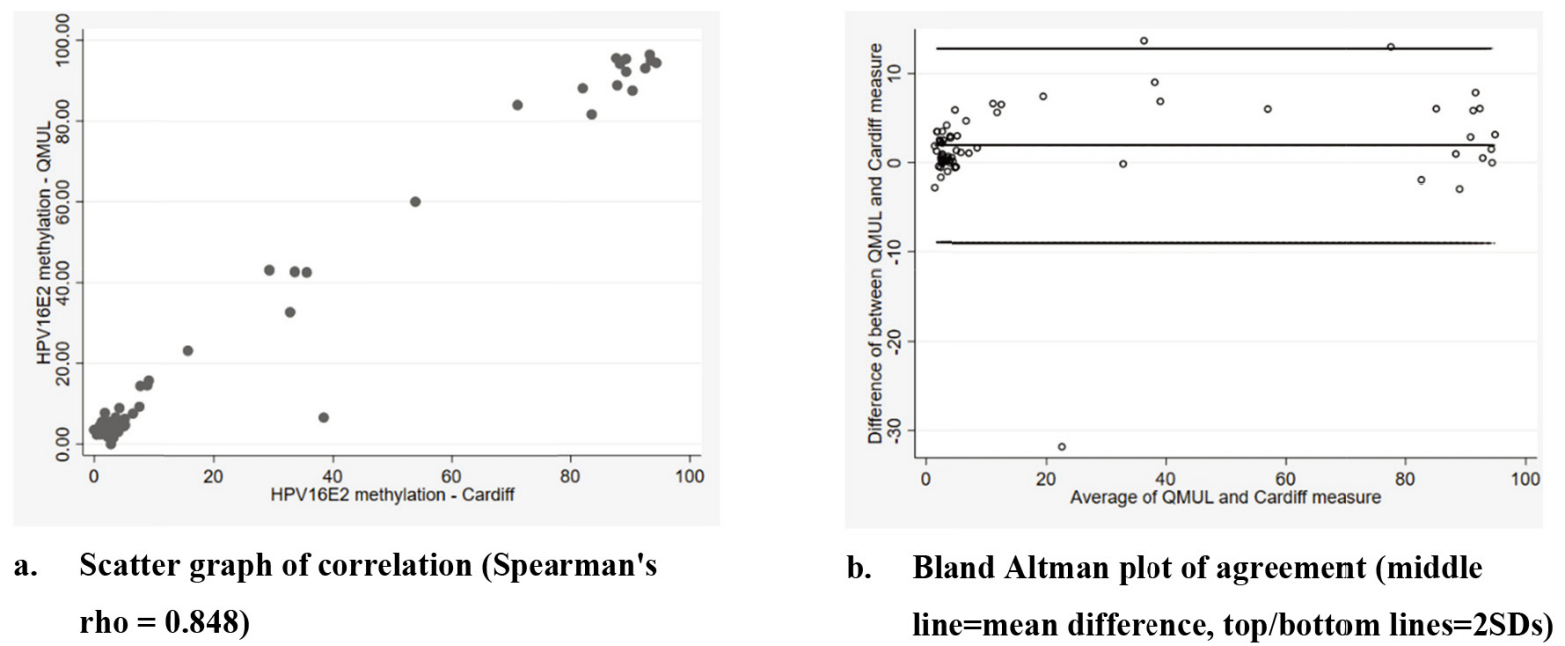
HPV16E2 methylation detection by laboratory.

## 3. Results

### 3.1 Distribution of DNA methylation markers by treatment group

Table 1 shows the distribution of pre-treatment DNA methylation biomarkers tested in the two treatment groups. Of all the patients randomised into the RT3VIN trial, 65/89 (73.0%) of patients in the cidofovir arm, and 67/91 (73.6%) in the imiquimod arm, had both a baseline sample and response assessment available; these 132 samples were used for the current study. HPV positivity and methylation assay results were reasonably well balanced between treatment arms. It can be seen that HPV 18, 31 and 33 positivity were relatively low in this cohort and that therefore the S5 score is largely dependent upon *EPB41L3*, HPV 16 *L1 and L2*.

**Figure 2.**
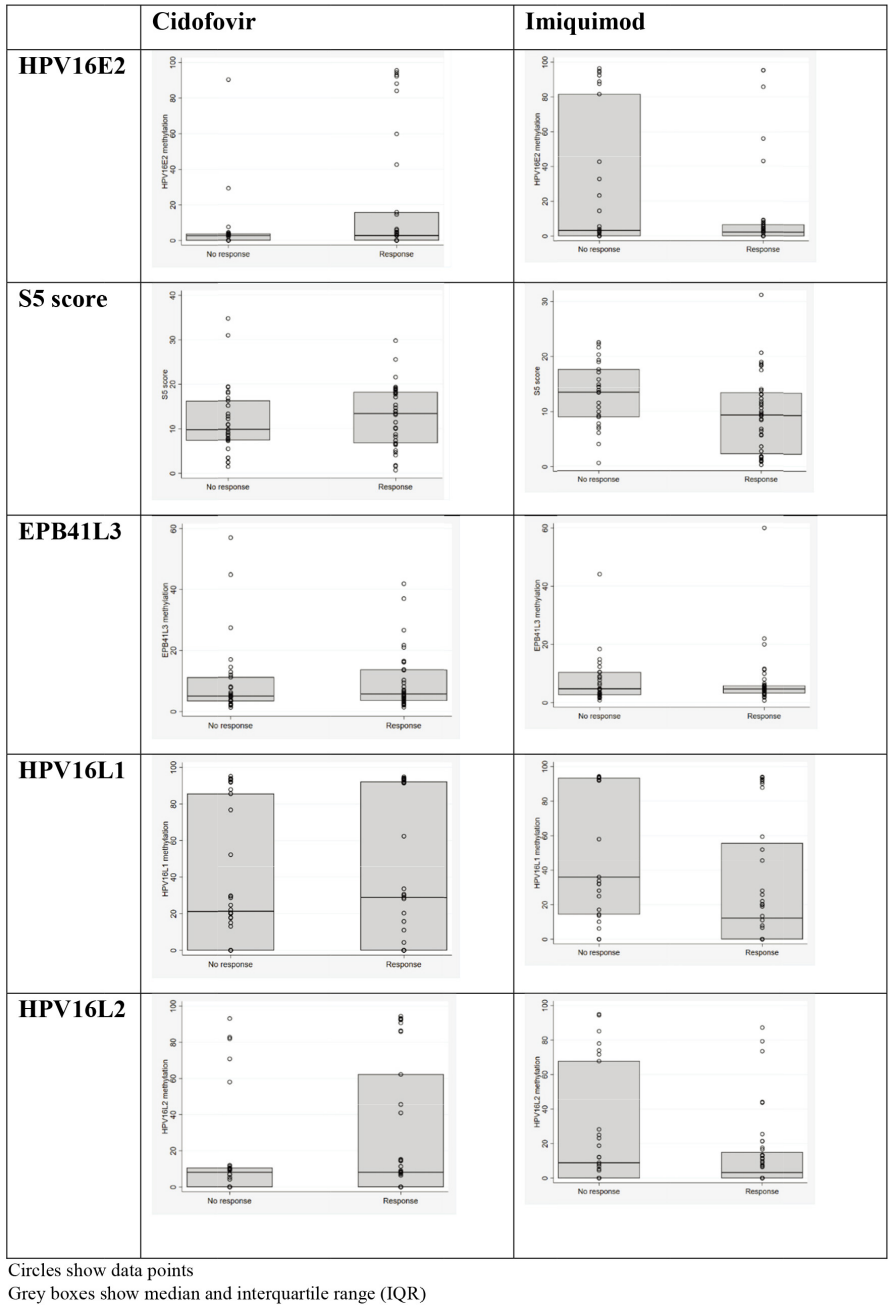
Distribution of methylation levels in responders and non-responders by trial arm.

**Table 3:** Response rates in methylation biomarker defined subgroups of the treatment groups

	n	Cidofovir		Imiquimod		p
HPV 16 $L\,1^{\text{𝑙𝑜𝑤}}$	67	16/33	(48.5)	26/34	(76.5)	0.018
*HPV16L1 = 0 & HPV16-ve*	*26*	*8/13*	*(61.5)*	*10/13*	*(76.9)*	*0.395*
*HPV16L1 = 0 & HPV16+ve*	*14*	*4/7*	*(57.1)*	*7/7*	*(100.0)*	*0.051*
*HPV16L1 > 0 & < 24.9*	*27*	*4/13*	*(30.8)*	*9/14*	*(64.3)*	*0.082*
HPV 16 $L\,1^{\text{ℎ𝑖𝑔ℎ}}$	65	19/32	(59.4)	14/33	(42.4)	0.172
HPV 16 $E\,2^{\text{𝑙𝑜𝑤}}$	85	21/46	(45.7)	24/39	(61.5)	0.144
*HPV16E2 = 0 & HPV16-ve*	*26*	*8/13*	*(61.5)*	*10/13*	*(76.9)*	*0.395*
*HPV16E2 = 0 & HPV16+ve*	*33*	*9/17*	*(52.9)*	*9/16*	*(56.3)*	*0.849*
*HPV16E2 > 0 & < 4*	*26*	*4/16*	*(25.0)*	*5/10*	*(50.0)*	*0.192*
HPV 16 $E\,2^{\text{ℎ𝑖𝑔ℎ}}$	47	14/19	(73.7)	16/28	(57.1)	0.247
HPV 16 $E\,2^{\text{ℎ𝑖𝑔ℎ}}$**or** HPV 16 $L\,1^{\text{ℎ𝑖𝑔ℎ}}$	76	21/36	(58.3)	21/40	(52.5)	0.610
HPV 16 $E\,2^{\text{𝑙𝑜𝑤}}$**and** HPV 16 $L\,1^{\text{𝑙𝑜𝑤}}$	56	14/29	(48.2)	19/27	(70.4)	0.093
HPV 16 $E\,2^{\text{ℎ𝑖𝑔ℎ}}$**and** HPV 16 $L\,1^{\text{ℎ𝑖𝑔ℎ}}$	36	12/15	(80.0)	9/21	(42.9)	0.026
HPV 16 $E\,2^{\text{𝑙𝑜𝑤}}$**or** HPV 16 $L\,1^{\text{𝑙𝑜𝑤}}$	96	23/50	(46.0)	31/46	(67.4)	0.035

HPV 16 $E\,2^{\text{𝑙𝑜𝑤}}$ is < 4% and HPV 16 $E\,2^{\text{ℎ𝑖𝑔ℎ}}$ is ⩾ 4%. HPV 16 $L\,1^{\text{𝑙𝑜𝑤}}$ is < 24.9% and HPV 16 $L\,1^{\text{ℎ𝑖𝑔ℎ}}$ is ⩾ 24.9%. All p values are unadjusted.

**Figure 3.**
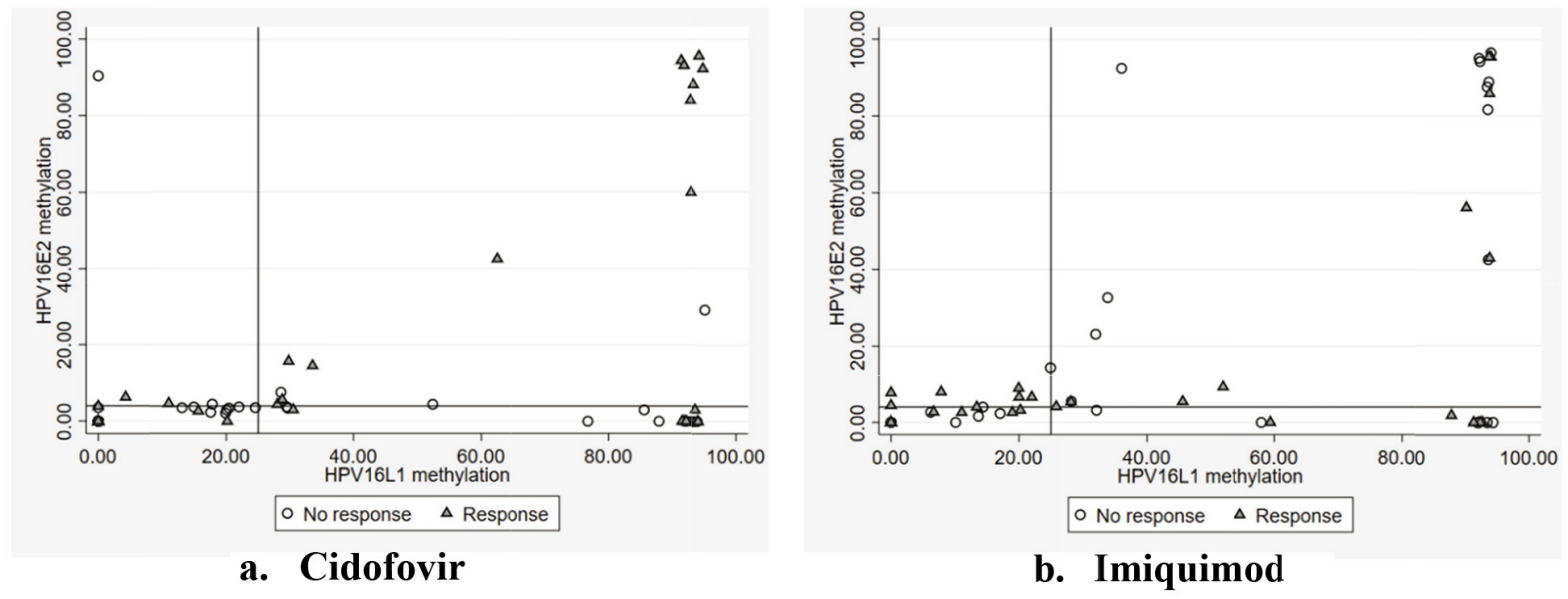
Scatter plot of HPV 16 *L1* and HPV 16 *E2* methylation by treatment group.

### 3.2 HPV 16 E2 methylation by assay

HPV 16 E⁢2 methylation level was assessed in both Cardiff and QMUL laboratories. Of the 132 patients included in this analysis, 63 had E⁢2 methylation detection in both laboratories between which there was both good correlation (Spearman’s rho = 0.848, Fig. 1a) and good agreement (Fig. 1b) apart from one outlier (6.61 in QMUL, 38.41 Cardiff). 10 had E⁢2 methylation detected in QMUL but not Cardiff (8 due to failed QC, 2 due to no HPV 16 detected). 58 patients did not have any E⁢2 methylation detected in either the Cardiff or QMUL laboratory. One patient had E⁢2 methylation detected in Cardiff but not in QMUL.

### 3.3 Biomarkers of response

Using the data from the QMUL laboratory, Table 2 and Fig. 2 show the distribution of HPV factors (after imputing missing values as 0) by response in each treatment group. There was some evidence (p< 0.2) that HPV negativity, HPV 16 negativity, low S5, low HPV 16 E⁢2, low HPV 16 L⁢1, and low HPV 16 L⁢2 were all associated with response in the imiquimod group. However, after correction for false discovery, only HPV 16 L⁢1 was significant with lower levels in the responders (median: 12.2; IQR: 0–55.7) than the non-responders (median: 36.0; IQR: 14.4–93.4) (z= 2.714, p= 0.006).

Although HPV 16 E⁢2 methylation, as measured in the QMUL laboratory, was higher in the cidofovir responders and lower in the imiquimod responders (consistent with previous findings), it was not identified as a significant marker of response in either treatment group. However, a sensitivity analysis that excluded samples with missing values (imputed as 0), showed the same results for HPV 16 E⁢2 methylation as measured in the Cardiff laboratory and reported elsewhere [8] i.e. significantly higher E2 methylation in the responders (median: 15.1, IQR: 4.5–88.1, n= 18) versus non-responders (median: 3.6, IQR: 3.4–4.4, n= 17) (z=-2.838, p= 0.005) in the cidofovir group and lower (albeit not significantly) HPV 16 E⁢2 methylation in the responders (median: 6.6, IQR: 4.0–9.2, n= 21) versus non-responders (median: 32.7, IQR: 4.0–88.8, n= 17) (z= 1.248, p= 0.212) in the imiquimod group.

### 3.4 HPV 16 L1 methylation as a biomarker for response with imiquimod

A ROC curve of HPV 16 L⁢1 methylation in predicting response in the imiquimod group (AUC = 0.694) found that the optimum cut point was ≥ 24.9 (HPV 16 $L\,1^{\text{ℎ𝑖𝑔ℎ}}$) which gave a sensitivity of 70.4% and specificity of 65.0%. Within the imiquimod group, a logistic regression showed that those with HPV 16 $L\,1^{\text{𝑙𝑜𝑤}}$ had an odds ratio of 4.31 (95% CI: 1.38–14.59; p= 0.009) of being a responder compared to those with HPV 16 $L\,1^{\text{ℎ𝑖𝑔ℎ}}$. This effect size was consistent in a multivariable analysis that included disease focality and whether or not the treated disease was first presentation or recurrent disease (OR = 4.26; 95% CI: 1.34–14.9; p= 0.0110).

### 3.5 Combining HPV 16 L1 and HPV 16 E2 methylation

An HPV 16 E⁢2 methylation level of 4% (HPV 16 $E\,2^{\text{𝑙𝑜𝑤}}$ is < 4% and HPV 16 $E\,2^{\text{ℎ𝑖𝑔ℎ}}$ is ⩾ 4%) has been previously shown [8] to define subgroups of patients who respond better to one topical treatment than the other. Figure 3 shows the correlation between HPV 16 L⁢1 and HPV 16 E⁢2 methylation and Table 3 shows the response with imiquimod and cidofovir for different combinations of HPV 16 L⁢1 and HPV 16 *E2 methylation*. We found that combining HPV 16 L⁢1 and HPV 16 E⁢2 may be more useful than using either biomarker on its own to define a subgroup of patients who may do better with one treatment than the other. Patients with both HPV 16 $L\,1^{\text{ℎ𝑖𝑔ℎ}}$**and** HPV 16 $E\,2^{\text{ℎ𝑖𝑔ℎ}}$ (36/132 (27.3%)) had a higher response with cidofovir (12/15 (80.0%)) than with imiquimod (9/21 (42.9%)) (p= 0.026). In contrast, patients displaying HPV 16 L1${}^{\text{𝑙𝑜𝑤}}$**or** HPV 16 E2${}^{\text{𝑙𝑜𝑤}}$ or no methylation result, had a higher response rate with imiquimod: 23/50 (46.0%) vs 31/46 (67.4%) (p= 0.035).

### 3.6 Sensitivity analyses exploring the validity of imputing missing values with zero

As described in the methods, in all the above analyses we imputed missing methylation values as 0 and included them in the HPV 16 $E\,2^{\text{𝑙𝑜𝑤}}$ or HPV 16 $L\,1^{\text{𝑙𝑜𝑤}}$ group. Sensitivity analyses shown in Table 3 explored the response to treatment in those patients who had missing values for methylation (HPV16E2 = 0, HPV16L1 = 0) and those who had HPV16 genotype detected (HPV16+ve) or not (HPV16-ve) and those who had low but non-zero (not missing) values of methylation. We noticed that more patients with HPV 16 $E\,2^{\text{𝑙𝑜𝑤}}$ methylation responded better to imiquimod than cidofovir in all subgroups albeit less so in the HPV16E2 = 0 and HPV16+ve subgroup. In the HPV 16 $L\,1^{\text{𝑙𝑜𝑤}}$ methylation group, response was consistently better with imiquimod even in the HPV16L1 = 0 & HPV16+ve subgroup (although the numbers were small).

## 4. Discussion

Cidofovir and imiquimod are topical treatments for vulval intraepithelial neoplasia; when used individually both treatments give complete response in approximately 50% of patients. HPV E2 methylation has been identified as a potential biomarker predictive of response to treatment, which could enable a more personalised approach with increased likelihood of efficacy [8]. A current limitation of the HPV E2 methylation assay is a high failure rate (approximately 50%). In this work, we demonstrated the problem of high failure rate could be overcome by combining HPV16 E⁢2 with HPV16 L⁢1 methylation; patients with HPV 16 $L\,1^{\text{ℎ𝑖𝑔ℎ}}$**and** HPV 16 $E\,2^{\text{ℎ𝑖𝑔ℎ}}$ respond better to cidofovir (80% response rate) and all others (including those with missing methylation for either marker) respond better to imiquimod (67% response rate). This suggests that the overall response rate of 50% with topical treatment could be improved to 71% with personalised treatment based on HPV methylation.

The assessment of HPV 16 E⁢2 methylation was broadly consistent between two laboratories. The QMUL analysis generated data for an additional ten patients; two of these were accounted for by HPV methylation assays being run on the full baseline cohort of patients rather than just those found to have HPV 16 present using the HPV 16 E⁢6 PCR and PapilloCheck assay. Inevitably, HPV detection assays will vary in their detection rates based on the target region of the virus they focus on and viral integration rates [17]. By applying the methylation assay to all baseline samples, it is not surprising that some additional HPV positive cases were identified. The eight other additional cases detected in QMUL were likely due to differences in the assay procedures used between the labs, including differing quality assurance parameters. However, this work provides validation of this method of determining methylation (pyrosequencing of the E2 ORF and HPV16 L1 gene using a Qiagen PyroMark Q96 ID system) and demonstrates that it can be successfully applied in multiple laboratories.

It is noted that the overall HPV prevalence in our cohort (86.4%) is somewhat higher than many studies quoted in the literature, including a systematic review of 2,764 cases of VIN (76.3%) published in 2017 [1]. However, this systematic review also describes a wide range of prevalence (0%–100%) and a more recent trial in the same population found a prevalence of 89.7% [18]. The high prevalence rate seen in our study may be due to the application of two HPV detection assays that target different regions of the HPV genome (E6 PCR targets E6 and PapilloCheck targets E1) which is not a common approach in many of the published studies.

To make this biomarker clinically useful, an approach to those patients generating no methylation (missing methylation) result is required. In this analysis we treated missing methylation values as “zero” i.e. low. This may not be biologically accurate since they may represent cases with high levels of viral integration, a feature typically associated with higher levels of methylation. However, we found the same pattern of better response with imiquimod in patients who were HPV 16 positive with missing methylation and in those patients who were HPV 16 negative with missing methylation. Consequently, the data support giving imiquimod to all patients with low and missing methylation, and cidofovir to those with both HPV 16 $E\,2^{\text{ℎ𝑖𝑔ℎ}}$**and** HPV 16 $L\,1^{\text{ℎ𝑖𝑔ℎ}}$; hence these biomarkers can be used to make a pragmatic treatment choice for all patients. These findings need to be validated in an external cohort of patients treated with imiquimod (which is a treatment modality currently used in clinical practice). If successful, then a randomised clinical trial is planned in which patients who are HPV 16 $E\,2^{\text{ℎ𝑖𝑔ℎ}}$**and** HPV 16 $L\,1^{\text{ℎ𝑖𝑔ℎ}}$ are randomised to either imiquimod or cidofovir.

S5 was not a significant marker of response in either imiquimod or cidofovir patients. The S5 classifier was initially designed as a triage tool for detection of CIN and cervical cancer and although it uses HPV and host methylation to do this, it was not designed as a biomarker to predict response to treatment. We noticed that the methylation levels of *EPB41L3* were low (5–10%) in VIN patients as opposed to cervical precancer (10–20%) or cancer patients (above 20%) [19]. It is possible that any discriminatory power of the S5 classifier was diluted by inclusion of *EPB41L3* which does not seem to differentiate responses in this population. This may reflect differences in the natural history and pathobiology of VIN and CIN.

HPV L1 methylation is a component of the S5 classifier and this study demonstrated that low HPV 16 L⁢1 methylation could predict response to treatment in the imiquimod group and that a cut off of ≥ 24.9 gave the optimum sensitivity and specificity. Combining HPV 16 E⁢2 and HPV 16 L⁢1 is better than using either marker alone for identification of patients who are likely to respond better to cidofovir. This may be because the HPV 16 $E\,2^{\text{ℎ𝑖𝑔ℎ}}$**and** HPV 16 $L\,1^{\text{ℎ𝑖𝑔ℎ}}$ more accurately measures “high methylation” by excluding those with borderline or mismatching results (low in one and high in the other).

Hypotheses to explain why high methylation is associated with response to cidofovir, and low methylation is associated with response to imiquimod, have previously been described [20]. These include that cidofovir may act as a demethylating agent. This is speculative but is consistent with cidofovir being a nucleoside analogue with similar structure to the established demethylating agent decitabine (used in the treatment of myelodysplastic bone conditions). This possibility is supported by a study of cases of failed cidofovir treatment in recurrent respiratory papillomatosis (caused by HPV 11), which correlated treatment failure with uniformly low levels of methylation [12]. Alternatively, E2 and L1 methylation may be surrogate markers of another relevant process, e.g. they may be associated with more advanced infections with lower levels of p53 protein. Although E6/E7 expression to some level is present at all times in an HPV infected cells independent of the presence of a ‘dormant’ infection or a disease causing infection, it is broadly accepted that it is deregulated E6/E6 expression that contributes to the development of a transforming (i.e. malignant potential) infection through the ubiquitination of p53/pRb rendering it dysfunctional. E2 DNA methylation is widely accepted as one means by which, E6/E7 gene expression becomes deregulated and consequently p53/pRb dysfunctional. This would be consistent with the suggestion that the selectivity of cidofovir for transformed cells is due to the absence, or perturbation, of normal DNA repair pathways associated with dysfunctional p53 mediated signalling [21]. Cidofovir has been shown to generate double-stranded breaks in cellular DNA, which can be repaired in normal cells, but not in tumour cells [22]. In HPV-infected cells, the level of p53 is reduced through ubiquitination and proteosomal degradation mediated by the HPV E6 oncoprotein, expression of which can become deregulated as a result of HPV integration and/or HPV DNA methylation [23]. HPV integration and increased methylation could therefore be more common in cells that have lower levels of p53/pRb and may be more likely to respond to cidofovir. The correlation between increased methylation and response to cidofovir could therefore be because methylation is a surrogate marker of absent/low-level p53/pRb.

Contrary to the case with cidofovir, E⁢2 and L⁢1 methylation was lower in patients who responded to imiquimod. Again, it is difficult to be certain whether it is the level of HPV methylation per se that is important in the activity of imiquimod, or whether E2 methylation is a surrogate marker for another important process or viral state. Imiquimod acts as an immunomodulator by activating TLR7, which in turn, enhances the innate immune system by stimulating the synthesis of proinflammatory cytokines, especially IFNα, which enhance cell-mediated cytolytic activity against viral targets [24, 25, 26]. However, the enhanced host immune response needs direction in order to be effective and it is plausible that a proliferative HPV infection provides this direction. A proliferative infection exists when HPV is present in episomal form in the cytoplasm of the cell and therefore genomically intact, able to replicate and proliferate. It is typically associated with earlier stage disease and therefore lower levels of methylation. The level of HPV methylation is broadly accepted to reflect severity of disease in cervical disease [27], so it is possible that those patients with low levels of methylation have persistent proliferative HPV infection that is able to direct the immunomodulatory effect of imiquimod.

In conclusion, this study refines the use of assessment of HPV methylation as a potential predictive marker in treatment in VIN for all patients from whom a sample is taken. It demonstrates that reproducibility of this method of assessment of methylation, that addition of human genes adds little to the predictive power of the marker panel, and that high levels of HPV gene methylation are consistently associated with good response to treatment with cidofovir and that low or missing levels are associated with good response to treatment with imiquimod. These findings justify validation in a prospective trial.
